# The effect of the intercellular adhesion molecule-1 and glycated haemoglobin on the management of diabetic neovascular glaucoma

**DOI:** 10.22336/rjo.2024.25

**Published:** 2024

**Authors:** Olga Volodymyrivna Guzun, Oleg Serhiyovіch Zadorozhnyy, Liudmyla Mykolayivna Velychko, Oleksandra Viktorivna Bogdanova, Lilia Gheorghe Dumbrăveanu, Vitalie Valeriu Cuşnir, Andrii Rostyslavovich Korol

**Affiliations:** * SI “The Filatov Institute of Eye Diseases and Tissue Therapy of the National Academy of Medical Sciences of Ukraine”, Odesa, Ukraine; ** “Nicolae Testemiţanu” State University of Medicine and Pharmacy, Chişinău, Republic of Moldova

**Keywords:** proliferative diabetic retinopathy, neovascular glaucoma, transscleral cyclophotocoagulation, intercellular adhesion molecule-1, glycated haemoglobin

## Abstract

**Introduction:** The study hypothesizes that some patients with diabetic neovascular glaucoma (NVG) do not fully respond to transscleral (TSC) cyclophotocoagulation (CPC) due to significant inflammation and insufficient glucose control.

**Objective:** The study aimed to determine the effect of baseline blood levels of intercellular adhesion molecule-1 (ICAM-1) and glycated haemoglobin (HbA1c) on the management of patients with diabetic NVG by TSC CPC.

**Methods:** This open prospective study included 70 diabetic patients (75 eyes; aged Ме 63.0 years) with painful NVG and 20 healthy individuals (aged Ме 61.5 years) as an immunological control. All patients underwent TSC СPC with a diode laser. Baseline HbA1c levels and ICAM-1 expression in blood samples were determined. Follow-up was 12 months.

**Results:** One month after TSC CPC, IOP decreased by 28% compared to baseline. The effectiveness of laser treatment after 12 months of follow-up was 63% with IOP decrease by 46%. In patients with NVG, the initial level of ICAM-1 was 2.5 times higher than in the control group. Patients who did not fully respond to the first TSC CPC (30 eyes) and required additional laser procedure, had high initial HbA1c (9.5%) and high expression values of the ICAM-1 (609.0 cells/μL).

**Conclusions:** Repeated procedures of TSC CPC at high IOP in diabetic patients with NVG are associated with high initial values of expression of ICAM-1 in peripheral blood and high HbA1c. The strategy of management of patients with diabetic NVG should be aimed at intensive glucose control and local anti-inflammatory treatment.

**Abbreviations:** PDR = proliferative diabetic retinopathy, DR = diabetic retinopathy, NVG = neovascular glaucoma, TSC CPC = transscleral cyclophotocoagulation, ICAM-1 = intercellular adhesion molecule-1, HbA1c = glycated haemoglobin, IOP = intraocular pressure

## Introduction

Proliferative diabetic retinopathy (PDR) is one of the leading causes of secondary neovascular glaucoma (NVG). The pathophysiology of PDR includes retinal microvascular damage and ischemia, resulting in the formation of new blood vessels of the retina, as well as neovascularization of the iris and trabecular meshwork, and leading to the subsequent development of neovascular glaucoma (NVG). NVG is the terminal stage of the disease and is accompanied by extremely high intraocular pressure (IOP) and severe ocular pain [**1**]. This complication is extremely challenging to manage.

Some studies support the view that subclinical inflammation favors the development of PDR. Subclinical inflammation supports the damage of microvessels in ocular tissues, with increased permeability and the appearance of neovascularization. Biomarkers of neuroinflammation, neurodegeneration, and vasculopathy are found in intraocular fluids, and their concentration changes at different stages of diabetic retinopathy (DR). Abnormal metabolic pathways cause the release of proangiogenic and inflammatory factors. These cytokines induce the expression of intercellular adhesion molecule-1 (ICAM-1, CD54). ICAM-1 is a key regulator of many important cellular functions in inflammatory conditions. It was found that with the DR progression, the levels of inflammatory mediators and the number of adhesion molecules such as ICAM-1 and VCAM-1 increase [**2**-**4**]. Infiltration of inflammatory cells into ischemic tissue through the endothelial cells of vessels contributes to the development of NVG [**5**,**6**]. 

Transscleral (TSC) cyclophotocoagulation (CPC) is widely used to reduce IOP in NVG patients. The mechanism of the therapeutic action of this procedure consists in reducing the production of intraocular fluid by the ciliary body. TSC CPC allows to achieve up to 88.6% success in the treatment of patients with glaucoma [**7**], however, in many patients, the IOP does not decrease sufficiently and they need repeated treatment courses. 

The hypothesis presented in the current study suggests that some patients with diabetic NVG may not experience a significant IOP reduction after undergoing TSC CPC. This may indicate the involvement of additional factors that influence IOP, leading to the need for additional laser treatment. We based our assumptions on the presence of pronounced inflammation and uncontrolled diabetes.

## Aim

The study aimed to determine the effect of baseline levels of intercellular adhesion molecule-1 (ICAM-1) and glycated haemoglobin (HbA1c) in the peripheral blood on the management of patients with diabetic NVG by TSC CPC.

## Materials and methods

*The studied population*

An open prospective cross-sectional study was conducted at the Filatov Institute of Eye Diseases and Tissue Therapy, a state institution affiliated with the National Academy of Medical Sciences of Ukraine. The study protocol adhered to the principles of the Declaration of Helsinki and received approval from the local bioethics committee. All study participants provided written informed consent.

The study participants were 70 patients (75 eyes) with diabetes mellitus (DM) types 1 or 2 and painful NVG. As an immunological control, data from 20 healthy individuals were used, in whom the expression of intercellular adhesion molecule-1 on lymphocytes from peripheral blood was determined.

**Fig. 1:**
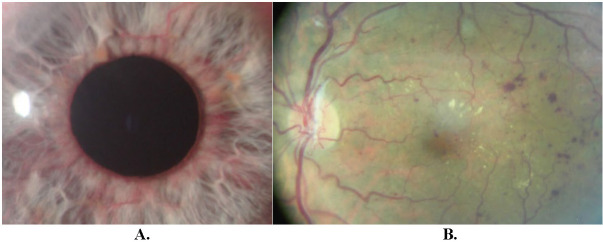
Patient T. Secondary diabetic NVG of the left eye, IOP 42 mmHg. **A.** anterior segment of the eye, neovascularization of the iris; **B.** fundus, retinal and optic disc neovascularization, haemorrhages, exudates

**Fig. 2:**
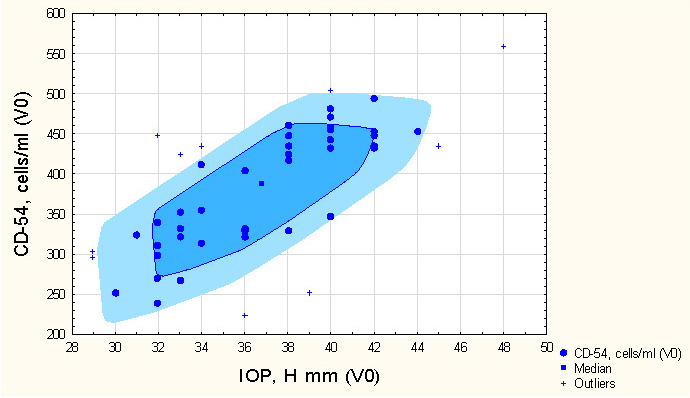
The expression level of the CD-54 molecule on peripheral blood lymphocytes in patients with NVG, based on initial IOP

Inclusion criteria for the study were: (1) diabetic NVG (**Fig. 1 A, B**), (2) uncontrolled IOP of 30 mmHg or higher, and (3) the presence of ocular pain. Exclusion criteria: (1) non-diabetic secondary neovascular glaucoma; (2) inability to perform TSC CPC, and (3) absence of ocular pain.

*Types of outcome measures*

The primary outcomes were: mean IOP decrease from initial values; final IOP ≤ 18 mmHg and one or more of 1) ≥ 20% reduction of IOP or 2) discontinuation of at least two hypotensive medications; need for additional TSC CPC sessions [**8**].

The secondary outcomes included: best corrected visual acuity (BCVA) (at a control examination after 12 months); painlessness (at all time points after TSC CPC); and total number of topical and systemic hypotensive medications.

*Study visits*

The initial visit (V0) was scheduled on the day before the laser procedure, followed by 1 month (V1), 3 months (V3), 6 months (V6), and 12 months (V12). Visual acuity, IOP (Goldmann tonometry), medication amount, presence of ocular pain, and clinical signs of inflammation were evaluated at each moment. All patients underwent biomicroscopy, ophthalmoscopy, and gonioscopy. 

*Laboratory tests*

ICAM-1 (CD54) expression in baseline blood samples was determined on peripheral blood lymphocytes using an immunocytochemical peroxidase-anti-peroxidase (PAP) technique and monoclonal antibodies (The R.E. Kavetsky Institute of Experimental Pathology, Oncology and Radiobiology of the National Academy of Sciences of Ukraine) in 42 patients. The main stages of this technique: obtaining a lymphocyte suspension by centrifugation on a Ficoll gradient (density 1.076 g/cm3, Simesta, Ukraine); double cell purification by centrifugation and preparation of smears; sequential application of monoclonal antibodies of a certain specificity, rabbit serum, and PAP complex to the smear. Microscopy (Euromex BioBlue microscope, Holland) with counting lymphocytes in the smear containing horseradish peroxidase per 100 free cells. 

HbA1c levels were measured by high-performance liquid chromatography (automatic analyzer BIO-RAD D10, Bio-Rad Laboratories, Inc., USA) from whole blood in all patients.

*Protocol for TSC CPC*

According to the standard technique, TSC СPC was performed with a diode laser Vitra 810 (Quantel Medical, France). The laser power ranged from 850 to 1500 mW (Me 1100 mW), the exposure time was 1.5-2 seconds, and the quantity of laser spots was 22 on average. The necessity for repeated TSC CPC was assessed in all patients at each follow-up visit. Repeated laser treatment was carried out while maintaining high IOP values.

*Statistical analysis*

Data processing was performed using the Statistica program (version 10.0, StatSoft Inc., USA). The Shapiro-Wilk test was used to check the normality of the continuous data distribution. Data were presented as mean (M) and standard deviation (SD), as well as median (Me) and Q 25% and Q 75% quartiles (Q 25%-75%). We used the Mann-Whitney U-test for pairwise comparison of two independent samples, and to check the equality of the medians of several samples - the Kruskel-Wallis test. The Wilcoxon T-test was used for repeated intragroup comparisons (V0, V1, V3, V6 and V12). We used Spearman’s non-parametric rank correlation coefficient to study the association between traits (rs). The critical level of significance during statistical hypothesis testing was assumed to be equal to p < 0.05. Multiple regression analysis was performed to identify the necessity for a repeated course of TSC CPС depending on clinical and laboratory parameters. Probable cumulative risk (Cox regression) for a repeated TSC CPС necessity was determined based on initial parameters. 

## Results

Seventy DM patients (75 eyes) with painful NVG associated with PDR were examined and treated. The age (Ме (Q 25%-75%)) of the NVG patients was 63.0 years (56-68), and the subjects of the control group - 61.5 years (56,5-65). The baseline demographic and clinical characteristics of patients detected at the initial visit (V0) are presented in **Table 1**.

**Table 1:** Demographic and clinical characteristics of patients with NVG

Parameters	Value
	Ме (Q 25%-7 5%) or n (%)
Age of patients with NVG, years	63.0 (56-68)
Male/Female, n (%)	32 (43%)/43 (57%)
Number of patients with NVG of both eyes, n (%)	5 (7%)
Spherical equivalent of refraction, Dptr	0.75 (-0.25-+1.25)
The axial length of the eye, mm	22.8 (22.4-23.4)
IOP, mmHg	36.0 (33-40)
Number of hypotensive medications, n	3.0 (2-3)
Number of eyes after glaucoma surgery, n (%)	25 (33%)
Number of eyes with cataract/pseudophakia, n (%)	51 (68%)/24 (32%)
Number of eyes after panretinal photocoagulation: yes/no, n (%)	29 (39%)/46 (61%)
Number of eyes after anti-VEGF therapy: yes/no, n (%)	17 (23%)/58 (77%)
BCVA	0.3 (0-0.02)
0, n (%)	26 (35%)
0.001-0.2, n (%)	49 (65%)
Bullous dystrophy of the cornea, n (%)	12 (16%)
Corneal oedema, n (%)	17 (24%)
Hyphema/vitreous haemorrhage, n (%)	11 (15%)/6 (8%)
Total eyes of patients with DM, n (%)	75 (100%)
type 1, n (%)	22 (29%)
type 2, n (%)	53 (71%)
Duration of DM, years	11.0 (7-15)
HbA1с, %	7.8 (7.2-8.6)
Expression of CD-54 molecule in patients with NVG, n = 42: cell/μL	545.0 (386.0-610.5)
(%)	30.0 (24-34)
Control group, n = 20: cell/μL	216.0 (187-234)
(%)	14.0 (12-17)
Cardiovascular pathology: yes/no, n (%)	48 (68.6%)/22 (31.4%)
Arterial hypertension, n (%)	36 (51.4%)
Chronic ischemic heart disease, n (%)	12 (17%)
Nephropathy/Neuropathy, n (%)	12 (17%)/8 (11%)
Note: Data are presented as median (Me), Q25%, and Q75% quartile (Q 25%-75%) or absolute number (n) and proportion in %.	

Initial IOP values at the first visit (V0) ranged from 29 to 48 mmHg (Me 36.0 mmHg) despite using an average of three hypotensive medications. In 50 cases (67%), patients received the maximal topical hypotensive therapy with an oral carbonic anhydrase inhibitor. 

The baseline level of expression of CD-54 on peripheral blood lymphocytes depending on the initial IOP in patients with NVG is presented in **Fig. 2**.

One month (V1) after TSC CPC, IOP decreased by 28% to 26.0 mmHg (min. 18.0-max. 38.0). After 12 months (V12), IOP decreased by 46% compared to baseline (**Table 2**).

**Table 2:** Dynamics of IOP and the number of hypotensive medications during 12 months of observation

Timepoint, (eyes)	IOP	IOP decrease from baseline, (%)	Hypotensive medications, (М ± SD)
	Ме (Q 25%-75%), mmHg		
V0 (n = 75)	36.0 (33-40)		3.0 ± 0.76
V1 (n = 74)	26.0 (21-32)**	(- 28%)	3.0 ± 0.92
V3 (n = 66)	21.0 (18-31)**	(- 42%)	2.0 ± 0.46
V6 (n = 66)	20.0 (18-21)**	(- 44%)	2.0 ± 0.56
V12 (n = 68)	19.5 (18-22)**	(- 46%)	1.0 ± 0.66*
Note: *р < 0.05, **р < 0.001 significance level from the initial data.			

The mean number of medications for NVG at 12 months post-TSC CPC (V12) was significantly reduced to 1.0 ± 0.66, and oral acetazolamide was discontinued in 43 of 50 cases (86% reduction). After undergoing TSC CPС, all patients experienced a complete regression of their ocular pain syndrome. Visual acuity did not change up to 6 months. On V6 (p < 0.05) and V12 (p < 0.05), an improvement of the BCVA was noted in 5 eyes due to the reduction of corneal oedema and resolution of intraocular haemorrhage. 

At the next stage, an analysis of cases requiring repeated laser treatment was carried out. Thus, one month (V1) after TSC CPC, 30 eyes (40%) still had IOP ≥ 30.0 mmHg, and they also had a recurrence of ocular pain syndrome. Therefore, these patients were assigned a repeated TSC CPC procedure. In ten eyes (13.3%), TSC CPC was carried out three times. On average, 1.51 ± 0.69 laser procedures were performed per patient with NVG. Clinical success was achieved in 47 (63%) cases after reintervention (12 months follow-up).

**Fig. 3:**
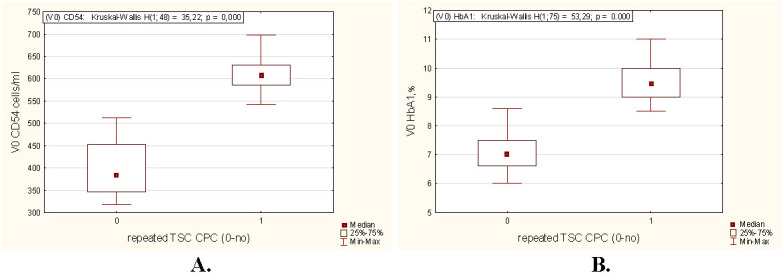
Distribution of initial laboratory test values (**А.** ICAM-1; **В.** HbA1c) depending on the need (1) or lack of need (2) for a repeated TSC CPC procedure in patients with NVG

In patients for whom TSC CPC was decided to be repeated at the V1 visit, the initial HbA1c values were 9.5%, and the expression values of the CD-54 molecule were 34.0%/609.0 cells/μL. Baseline ICAM-1 and HbA1c expression values were higher in patients who required a second TSC CPC procedure at visit V1, as shown in **Fig. 3 A, B**.

A multiple linear regression analysis was conducted to quantify the relationship between IOP and the initial blood levels of ICAM-1 (model 1) and HbA1c (model 2) to predict the necessity for a repeated TSC CPC (**Table 3**).

**Table 3:** The need for a repeat course of TSC CPC depending on the initial values of IOP, CD-54, and HbA1c (а multiple regression analysis)

Parameters	Model 1	Model 2
R2 adjusted	0.85	0.84
β (S); р		
V0 IOP	0.18 (0.08); р = 0.02	0.27 (0.07); р = 0.001
V0 CD-54	0.79 (0.08); р = 0.000	
V0 HbA1с	-	0.73 (0.07); р = 0.000
F	139.3; р = 0.000	128.6; р = 0.000
Note: R2 adjusted - is the adjusted coefficient of determination; β - standardized regression coefficient; S - errors of regression coefficients; F - is the calculated value of the F-criterion; p - is the probability of the null hypothesis for the F-criterion.		

The analysis results indicated a significant association between the necessity for a repeated TSC CPC and the assessed predictor variables (IOP and CD-54 in model 1 (R2 = 85%; F (2.45) = 139.3; p = 0.000) or IOP and HbA1c in model 2 (R2 = 84%; F (2.45) = 128.6; р = 0.000)) in both presented models.

**Fig. 4:**
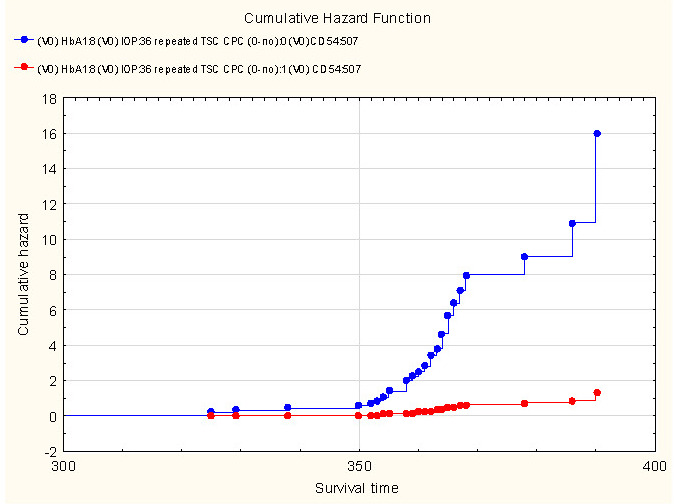
Cumulative risk plot. The relationship between the need for repeated TSC CPC and the values of HbA1c, CD-54, and IOP

The cumulative risk plot (Cox regression) demonstrated that an IOP of 36 mmHg and increased expression of CD-54 (from 507 cells/μl) and HbA1c (from 8%) increased the probable risk for a repeated TSC CPC (**Fig. 4**).

Direct correlations according to Spearman (p < 0.05) were found to be needed for repeated TSC CPC with CD54 (rs = 0,87), HbA1с (rs = 0,85), IOP (rs = 0,63), complications (rs = 0,61), DM duration (rs = 0,58), laser power (rs = 0,38), number of laser spots (rs = 0,34), and panretinal photocoagulation (rs = 0,26).

During the entire observation period, some complications were recorded. Subconjunctival haemorrhage was detected in 11 (14.6%) eyes in the one-week follow-up after treatment. Hyphema was observed in 8 (11%) eyes in three months. Corneal endothelial decompensation, photophobia, and lacrimation were reported in 14 (18.7%) cases with previous corneal pathology (corneal oedema and bullous dystrophy). Postoperative inflammation was observed in 28 (37%) eyes. These patients were prescribed topical corticosteroids (preservatives-free dexamethasone in single doses) 3 times per day for 2 weeks in combination with non-steroidal anti-inflammatory therapy (bromfenaс) 1 time per day for 4 weeks. No other serious complications were observed, including prolonged ocular hypotony, ocular phthisis, retinal or choroidal detachment, and decreased visual acuity.

## Discussion

The increase in pro-inflammatory mediators in DR patients highlights an important inflammatory shift associated with the disease [**9**]. Muni and colleagues showed that the progression of DR is associated with chronic subclinical inflammation with increased circulating levels of ICAM-1 [**4**]. Other authors showed that chronic subclinical inflammation is responsible for the vascular changes in DR. They noted that an increase in the expression of ICAM-1 and integrins on endothelial cells and leukocytes leads to diabetic leukostasis of the retina and disruption of the blood-retinal barrier [**10**,**11**]. The association of cell adhesion molecules and selectins with the development of microvascular disorders in DM patients is also confirmed [**12**,**13**]. Similarly, in our study of DM patients with NVG, the level of ICAM-1 was 2.5 times higher than in age-matched healthy volunteers. Moreover, this indicator in patients who did not fully respond to TSC CPC (and were given a repeated laser treatment) was 37% higher.

Jin et al. found a high increase in the progression rate of DR in patients with a baseline HbA1с level of over 6.4% [**14**]. Abnormalities in levels of fasting blood sugar and HbA1c also may be considered risk factors for NVG development [**15**]. The results of the presented study demonstrated that the need for a repeated procedure of TSC CPC in NVG patients was associated with higher preoperative expression levels of the specific inflammatory biomarker ICAM-1, with a higher level of HbA1с and high initial IOP values.

During a 6-month follow-up, Williams and colleagues found IOP reduction by 51% in 66% of cases and the following complications of TSC CPC: persistent ocular hypotony (8.8%), long-term inflammation (26%), reduction of BCVA ≥ 2 lines (17%), macular oedema (5%), corneal oedema (2.5%) and ocular phthisis (2.5%) [**16**]. In our study, in NVG patients after TSC CPC, prolonged inflammation was recorded in 37% of cases, decompensation of the corneal endothelium in 18.7%, and hyphema in 11%. However, complications may arise because of a general disease, for example, intravitreal haemorrhages, as well as late hyphema three months after TSC CPC (8 cases). Considering the current trend of performing TSC CPC in eyes with high visual functions, reducing the risk of complications in this patient category is particularly relevant [**17**].

Nabili and Kirkness used diode TSC CPC in the management of diabetic NVG refractory to drug therapy and found visual acuity deterioration in 30% of eyes, and phthisis in 25% of eyes [**18**]. Ramli and colleagues reported that the frequency of hypotony after TSC CPC was 39% [**19**]. The high incidence of hypotony in NVG patients after laser treatment is probably because in conditions of ischemia and inflammation, the intraocular fluid production is disturbed, and its outflow is also impaired due to the fibrovascular tissue of the drainage system. Diode TSC CPC can disrupt the balance between outflow resistance and fluid production due to postoperative ciliary body inflammation, which leads to hypotony. Possibly due to low laser energy usage for TSC CPC, our study did not underline visual functions, hypotony, and phthisis decrease. Thus, the use of lower laser energies for TSC CРC can lead to the risk of complications decrease with a sufficient level of laser exposure to the ciliary body to achieve a therapeutic effect [**20**]. Egbert et al. also found no significant difference in success (85%/87%) when using low-energy (energy 45.0 J, power - 1.5 W, exposure - 1.5 s, 20 laser spots) or high-energy (energy 62.5 J, power - 1.25 W, exposure - 2.5 s, 20 laser spots) of laser radiation [**21**].

In our study, absolute success after TSC CPC (12 months follow-up; an average 1.51 ± 0.69 laser procedures per patient) was observed in 63% (47/75) of cases with a decrease in IOP by 46% and in hypotensive medications by an average of 2 drugs. Other authors also showed a high success rate (from 58.5% to 86.7%) during one year of follow-up after TSC CPC (an average of 1.9 laser procedures per patient with NVG) without serious complications [**22**,**23**]. Elhusseiny et al. reported a 63.4% success rate of TSC CPC after 1 year, with a 36% IOP reduction and a reduction in therapy of 1.4 hypotensive medications [**24**]. The efficacy and safety of TSC CPC retreatment were reported by Kuchar et al., but they observed a small sample (only 3 patients) and demonstrated an increased success rate (15.8%) after retreatment [**25**]. Lim et al. reported on the effectiveness and safety of micropulse TSC CPC in eyes with advanced glaucoma [**26**]. In the Hooshmand study, repeated micropulse TSC CPC achieved a significant IOP reduction of almost 5 mmHg after 6 months (p < 0.002) without adverse effects. However, none of the patients with NVG were followed up [**27**]. In the current study, the second TSC CPC procedure was found to enhance the success rate in 13.3% of cases, but in 10 NVG patients IOP reduction was not achieved and a third laser session was carried out.

Cox regression analysis revealed that the probable cumulative risk of a repeated TSC CPC at high IOP in NVG patients was associated with an increase in initial values of HbA1c, as well as with the expression of ICAM-1. The coefficient of determination in the multiple regression analysis also showed that IOP, CD-54, and HbA1c influence the need for repeated TSC CPC. In a prior study of patients following TSC CPC, it was found that the reduction in IOP depends on the decrease in the level of the inflammatory biomarker ICAM-1 in the peripheral blood, against the background of long-term (3 months) local non-steroidal anti-inflammatory therapy with ketorolac [**28**]. 

We believe that to control intraocular inflammation (with elevated initial CD-54) in patients with diabetic NVG, it is appropriate to add topical steroids and non-steroidal anti-inflammatory drugs for treatment. The steroid dose should be reduced very slowly, reducing by one drop every 2-3 weeks in combination with ketorolac, which is also recommended by other authors [**29**]. In addition to controlling inflammation to reduce the risk of DR progression and decrease the need for repeated laser treatments for NVG, more intensive glucose control is recommended. After the stabilization of DM, we also suggested the possibility of using immunotherapy methods in patients with NVG and complications of DM: retinopathy, nephropathy, and neuropathy, which can improve their quality of life and correlate with the recommendations of other authors [**30**].

## Conclusions

The effectiveness of the diode TSC CPC in diabetic patients with NVG during 12 months of follow-up was 63%, with an IOP decrease of 46%. Repeated procedures of TSC CPC at high IOP in eyes with diabetic NVG are significantly associated with high initial values of ICAM-1 in peripheral blood and high glycated haemoglobin. The strategy of treatment of patients with diabetic NVG should be aimed at intensive glucose control and local anti-inflammatory treatment. 

**Conflict of Interest Statement**

The authors report no conflicts of interest. 

**Informed Consent and Human and Animal Rights Statement**

All participants signed a written informed consent. 

**Authorization for the use of human subjects**

Ethical approval: The study protocol was approved by the Ethics Committee of the SI “The Filatov Institute of Eye Diseases and Tissue Therapy of the National Academy of Medical Sciences of Ukraine” (protocol № 2, 05.02.2024), and was performed according to the principles of safety, ethical attitude and application of the rules of working with patients following the “Bioethical Regulations of the Declaration of Helsinki on the ethical regulation of medical research”, the Convention of the European Council on Human and Biomedical Rights and the relevant Laws of Ukraine.

**Acknowledgments**

None. 

**Sources of Funding**

No funding was received for this research. 

**Disclosures**

None.
